# Prevalence of tobacco smoking and its socioeconomic determinants: Tobacco smoking and its determinants

**DOI:** 10.1111/crj.13470

**Published:** 2022-01-21

**Authors:** Hamideh Mahdaviazad, Reza Foroutan, Seyed Masoom Masoompour

**Affiliations:** ^1^ Family Medicine Department, School of Medicine Shiraz University of Medical Sciences Shiraz Iran; ^2^ Student Research Committee Shiraz University of Medical Sciences Shiraz Iran; ^3^ Non‐Communicable Diseases Research Center Shiraz University of Medical Sciences Shiraz Iran

**Keywords:** cigarette smoking, hookah smoking, prevalence, socioeconomic status, tobacco use

## Abstract

**Introduction:**

The epidemic tobacco use is a public health concern worldwide. This study aimed to evaluate the prevalence of tobacco use and its socioeconomic determinants in the city of Shiraz, Iran.

**Methods:**

In total, 5873 adults aged 20 and older were included in this study from the city of Shiraz, Iran, from June to October 2015. The sampling was conducted using the stratified random sampling method. Active cigarette, hookah, and second‐hand smokers were labeled as tobacco users in this study. Past smokers and non‐smokers were labeled as non‐tobacco users. The participants' socioeconomic status (SES) was determined based on their self‐reported level of education, occupation, income, and residence.

**Results:**

In this study, 35.4% of the participants were tobacco users. The prevalence of active cigarette, active hookah, dual‐users, and secondhand smokers was 13.3%, 8.3%, 0.4%, and 13.4%, respectively. The prevalence of tobacco use was highest among individuals with primary education level (40.9%), manual jobs (46.4%), lowest income level (38.1%), and those living in the suburban areas (36.4%). In multivariate analysis, the most socioeconomic factors related to tobacco usage were lack of academic education, manual job, and low‐income level.

**Conclusions:**

Tobacco control efforts should be more focused on vulnerable groups of cigarette and hookah users in the southwest of Iran. Moreover, SES and reduction of health‐related disparities and inequality should be considered a crucial concern in this regard.

## 1 INTRODUCTION

The epidemic tobacco use (e.g., cigarette and hookah) is a public health matter worldwide.^1^, ^2^ Smoking is one of the significant modifiable risk factors of non‐communicable diseases (NCDs). According to Iran's health profile 2015, NCDs are accountable for more than three quarters of mortality in Iran.^3^

Although Iran has been a member of the Framework Convention on Tobacco Control (FCTC) since 2003, the rate of tobacco use and its adverse consequences are still high.^4^ Based on the report of the World Health Organization (WHO), 20% and 0.8% of Iranian men and women aged 15 and overuse tobacco on a daily basis, respectively.^5^ Based on the National Surveys of Risk Factor of Non‐Communicable Diseases (STEPS), the prevalence of cigarette and hookah use was estimated at 23.7% for men and 3.0% for women, of whom 20.2% of men and 0.8% of women were exclusive cigarette smokers, 2.7% and 2.2% were exclusive hookah users, and 0.6% and 0.01% smoked both cigarettes and hookah.^6^

The social determinants of health had a major role in fostering chronic diseases and disabilities.^7^, ^8^, ^9^ Similar to most of the developing countries, the socioeconomic status (SES) of people in Iran has caused striking health‐related inequalities among individuals with different income levels. Therefore, chronic diseases and behavioral risk factors are more prevalent among people with lower income and educational levels.^10^, ^11^ Previous studies have investigated the relationship between the prevalence and pattern of tobacco use and geographic distribution or form of use.^1^, ^6^, ^12^, ^13^, ^14^, ^15^ However, to the best of the authors' knowledge, no study has yet assessed the role of SES among Iranian adult tobacco users. Therefore, the present study aimed to evaluate the prevalence of cigarette and hookah smoking and its socioeconomic determinants among the adult population residing in the city of Shiraz, Iran, using data from Shiraz Adult Respiratory Disease Study, 2015 (SARDS).

## 2 METHODS

### 2.1 Setting and sampling

This population‐based study was conducted on the adult population of Shiraz, the capital city of Fars province in the southwest of Iran, from June to October 2015. According to the 2011 national census, the population of Fars province was 4.59 million, of which 1.7 million live in the city of Shiraz and its suburbs.^16^ The study was carried on 6109 non‐institutionalized inhabitants aged 20 and older from the nine municipal districts of Shiraz, Iran. The sampling was performed using a stratified random sampling method, and the study population was proportional to the municipal districts and strata population. The sample population consisted of 0.5% of all population aged 20 and older residing in Shiraz, Iran. The details of the Shiraz Adult Respiratory Disease Study (SARDS) methodology were depicted elsewhere.^17^ As a part of SARDS' study, after obtaining official permission to visit homes, the trained interviewers introduced themselves and explained the objectives of the study to the main members of the households. Subsequently, the eligible members were invited to complete the questionnaires. The interviewers helped participants to complete the questionnaires if they were unable to complete the questionnaires by themselves.

### 2.2 Definitions

In this study, active cigarette and active hookah smokers referred to those who smoked at least one cigarette or one hookah head per day at the time of the study, respectively. Those who simultaneously used both cigarettes and hookah were classified as “dual users.” “Second‐hand smokers” were non‐smoker individuals who frequently inhaled hookah or cigarette smoke at or outside their home. Individuals who stopped smoking cigarettes or hookah during the last 12 months without any relapse were considered “past smokers.” Those with no history of cigarette or hookah smoking during their lifetime were called “non‐smokers.” Those who were “active cigarette smoker,” “active hookah smoker,” “dual user,” or “second‐hand smoker” were labeled as “Tobacco users.” The aggregation of “Non‐smokers” and “Past smokers” were referred to as “Non‐tobacco users.”

All the participants were questioned about the daily quantity of tobacco usage per head, the number of cigarettes used per day, and the duration of smoking in years.

The individual SES was extrapolated from the individual's level of education, occupation, income, and residence (urban or suburban). Education level was categorized into illiterate, primary, secondary/high school, and academic. The current occupation of participants was asked through an open‐ended question, and the status of their job was categorized into non‐manual, manual, jobless, or unspecified. Persons without a paid job at the time of interview who were available and capable to work were considered jobless. The participants with “unspecified occupation” were those incapable of working, students, retired people, and those with unknown jobs. The total income of households during a year was categorized into four different ranges from <$3500 to >$7000 per year, considering the average income of all households in Iran.

### 2.3 Statistical analysis

The data were analyzed using the SPSS software (Chicago, Illinois; Version 15.0). The investigator (H.M.) double‐checked the data to reduce the chance of human error. Continuous variables were reported as mean and standard deviation, and categorical variables were reported as numbers and percentages. The means of two continuous variables were compared using an independent Student's *t* test. The frequencies of categorical variables were compared using the Chi‐Square test. All variables with a *p* value less than 0.05 in univariate analysis were entered into the multiple logistic regression model to estimate adjusted odds ratios (ORs) and 95% confidence intervals (CI). A *p* value less than 0.05 was considered statistically significant.

## 3 RESULTS

Out of 6109 individuals invited to this population‐based study, 5873 (96.14%) individuals were accepted and 236 (3.86%) persons refused to complete the interview process (non‐respondents). Demographic characteristics of non‐respondents were not significantly different from respondents.

The mean ± SD age of participants was 51.44 ± 14 years, and the majority of respondents (54.4%) were female. The mean ± SD of BMI was obtained at 26.15 ± 4.92. Among the participants, 41.7%, 55.5%, and 74.3% had primary‐school education, non‐manual job, and yearly income level of <$3500, respectively, and 96.6% lived in an urban area (Table 1).

**TABLE 1:** Sociodemographic characteristics of the participants—Shiraz Adult Respiratory Disease Study, 2015

Variables		Number %, mean ± SD
Age, years		51.44 ± 14
Gender	Men	2677 (45.6%)
	Women	3196 (54.4%)
BMI		26.15 ± 4.92
Education level	Illiterate	743 (12.7%)
	Primary	2440 (41.7%)
	Secondary	1657 (28.3%)
	Academic	1007 (17.2%)
Occupation	Manual	1345 (22.9%)
	Non‐manual	3262 (55.5%)
	Jobless	146 (2.5%)
	unspecified	1097 (18.8%)
Income, yearly	Less than 3500$	4343 (74.3%)
	3500 to 4750$	1116 (19.1%)
	5250 to 7000$	239 (4.1%)
	More than 7000$	148 (2.5%)
Residency	Suburban	198 (3.4%)
	Urban	5648 (96.6%)

### 3.1 Prevalence

Overall, the prevalence of tobacco use including cigarette, hookah, second‐hand smoke, or dual‐use among participants was 35.4% (*n* = 2073). Men had a higher rate of tobacco use than women (38.3% vs. 32.9%, *P* < 0.001) (Table 2). Totally, 13.3% of the participants (27.9% of men and 1.0% of women) were active cigarette smokers (*P* < 0.001). The prevalence of cigarette smoking reached its peak among men and women at the age range of 41–50 years (35.1%) and 20–30 years (1.5%), respectively (Table 3). The age at which men and women started smoking cigarettes was 29 and 36, respectively. Men smoked a higher number of cigarettes per day than women (14.3 vs. 10.2, *P* = 0.021).

**TABLE 2:** Prevalence of tobacco use by demographic and socioeconomic factors

Variables		Total (*n* = 5873)	Tobacco use (*n* = 1288)	Non‐tobacco use (*n* = 4585)	*P* value
Age (years), Mean ± SD		51 ± 14	50 ± 13	52 ± 14	<0.001
BMI (kg/m^2^), Mean ± SD		26.16 ± 4.9	25.93 ± 5.07	26.28 ± 4.8	0.011
Gender, *n*%	Men	2677 (45.6%)	1024 (38.3%)	1653 (61.7%)	<0.001
	Women	3196 (54.4%)	1052 (32.9%)	2144 (67.1%)	
Education level, *n*%	Illiterate	743 (12.7%)	256 (34.5%)	487 (65.5%)	<0.001
	Primary	2440 (41.7%)	999 (40.9%)	1441 (59.1%)	
	Secondary	1657 (28.3%)	564 (34%)	1093 (66%)	
	Academic	1007 (17.2%)	248 (24.6%)	759 (75.4%)	
Occupation, *n*%	Manual	1345 (23%)	624 (46.4%)	721 (53.6%)	<0.001
	Non‐manual	3262 (55.8%)	1070 (32.8%)	2192 (67.2%)	
	Jobless	146 (2.5%)	61 (41.8%)	85 (58.2%)	
	unspecified	1097 (18.8%)	317 (28.9%)	780 (71.1%)	
Income (yearly), *n*%	Less than 3500$	4343 (74.3%)	1655 (38.1%)	2688 (61.9%)	<0.001
	3500 to 4750$	1116 (19.1%)	311 (27.9%)	805 (72.1%)	
	5250 to 7000$	239 (4.1%)	69 (28.9%)	170 (71.1%)	
	More than 7000$	148 (2.5%)	30 (20.3%)	118 (79.7%)	
Residency, *n*%	Suburban	198 (3.4%)	72 (36.4%)	126 (63.6%)	0.763
	Urban	5648 (96.6%)	1995 (35.3%)	3653 (64.7%)	

**TABLE 3:** Prevalence of tobacco use stratified by cigarette and hookah in Iranian adults based on SARDS 2015

Characteristics	Tobacco use		Active cigarette smoker		Active hookah smoker		Dual use		Second‐hand smoker		
Men	Number	Prevalence (95% CI)	Number	Prevalence (95% CI)	Number	Prevalence (95% CI)	Number	Prevalence (95% CI)	Number	Prevalence (95% CI)	Total
20–30	106	40.8% (34.9–46.8)	31	11.9% (8.4_16.3)	38	14.6% (10.7–19.3)	3	1.2% (0.3–3)	34	13.1% (9.4–17.6)	260
31–40	167	48.8% (43.6–54.1)	111	32.5% (27.7_37.5)	30	8.8% (6.1–12.1)	6	1.8% (0.7–3.6)	20	5.8% (3.7–8.7)	342
41–50	282	42.3% (38.6–46.1)	234	35.1% (31.6_38.8)	32	4.8% (3.4–6.6)	3	0.5% (0.1–1.2)	13	2% (1.1–3.2)	666
51–60	240	39.3% (35.5–43.3)	202	33.1% (29.5_36.9)	21	3.4% (2.2–5.1)	2	0.3% (0.1–1)	15	2.5% (1.4–3.9)	610
older 60	229	28.8% (25.8–32.1)	170	21.4% (18.7_24.4)	20	2.5% (1.6–3.8)	1	0.1% (0–0.6)	38	4.8% (3.5–6.4)	794
Total	1024	38.3% (36.5–40.2)	748	28% (26.3_29.7)	141	5.3% (4.5–6.2)	15	0.6% (0.3–0.9)	120	4.5% (3.8–5.3)	2672
**Women**											
20–30	66	33.2% (26.9–39.9)	3	1.5% (0.4_4)	9	4.5% (2.3–8.1)	1	0.5% (0.1–2.3)	53	26.6% (20.9–33.1)	199
31–40	169	35.6% (31.4–40)	2	0.4% (0.1_1.3)	43	9.1% (6.7–11.9)	1	0.2% (0–1)	123	25.9% (22.1–30)	475
41–50	330	35.6% (32.6–38.7)	8	0.9% (0.4_1.6)	127	13.7% (11.6–16)	2	0.2% (0–0.7)	193	20.8% (18.3–23.5)	927
51–60	302	33.4% (30.4–36.6)	11	1.2% (0.6_2.1)	97	10.7% (8.8–12.9)	3	0.3% (0.1–0.9)	191	21.2% (18.6–23.9)	903
older 60	182	26.5% (23.3–29.9)	7	1% (0.5_2)	67	9.8% (7.7–12.2)	0	0% (0–0)	108	15.7% (13.2–18.6)	686
Total	1049	32.9% (31.3–34.5)	31	1% (0.7_1.4)	343	10.8% (9.7–11.9)	7	0.2% (0.1–0.4)	668	20.9% (19.6–22.4)	3190
**Both sexes**											
20–30	172	37.5% (33.1–42)	34	7.4% (5.3_10.1)	47	10.2% (7.7–13.3)	4	0.9% (0.3–2.1)	87	19% (15.6–22.7)	459
31–40	336	41.1% (37.8–44.5)	113	13.8% (11.6_16.3)	73	8.9% (7.1–11)	7	0.9% (0.4–1.7)	143	17.5% (15–20.2)	817
41–50	612	38.4% (36.1–40.8)	242	15.2% (13.5_17)	159	10% (8.6–11.5)	5	0.3% (0.1–0.7)	206	12.9% (11.4–14.6)	1593
51–60	542	35.8% (33.4–38.3)	213	14.1% (12.4_15.9)	118	7.8% (6.5–9.2)	5	0.3% (0.1–0.7)	206	13.6% (12–15.4)	1513
older 60	411	27.8% (25.5–30.1)	177	12% (10.4_13.7)	87	5.9% (4.8–7.2)	1	0.1% (0–0.3)	146	9.9% (8.4–11.5)	1480
Total	2073	35.4% (34.1–36.6)	779	13.3% (12.4_14.2)	484	8.3% (7.6–9)	22	0.4% (0.2–0.6)	788	13.4% (12.6–14.3)	5862

Overall, 8.3% of the population were active hookah smokers, and the prevalence of active hookah smoking was two times higher in women compared to men (10.8% vs. 5.3%, *P* < 0.001). The prevalence of hookah smoking reached the peak two decades earlier among men compared to women (age range 20–30 vs. 41–50 years). Among the old population, the prevalence of hookah smoking in women was nearly four times higher than men (9.8% vs. 2.5%). Dual smokers constituted only 0.4% (0.6% of men and 0.2% of women) of the entire study population. The prevalence of secondhand smoking was 13.4% in the entire population of participants, which was significantly higher among women (20.9%), compared to men (4.5%) (*P* < 0.001). The prevalence of second‐hand smoking reached the peak among women (26.6%) and men (13.1%) aged 20–30 years (Table 3).

### 3.2 Tobacco use and socioeconomic related factors

Tobacco users were slightly younger than non‐tobacco users (50.21 ± 13 vs. 51.78 ± 14; *P* < 0.001). The association between tobacco use and BMI was statistically significant. The BMI was lower among non‐tobacco users, compared (25.46 ± 5.25 vs. 26.34 ± 4.77; *P* < 0.001).

The prevalence of tobacco use was highest in participants with primary education (40.9%) than those with other educational levels (*P* < 0.001). The prevalence of tobacco use was higher among individuals with manual jobs (46.4%). The prevalence of tobacco use was significantly higher among the lowest income category (<$3500) than other income categories (38.1%; *P* < 0.001). Eventually, tobacco use was more prevalent among those living in urban areas compared to those living in suburban areas (36.4% vs. 35.3%). The demographic and socioeconomic characteristics of tobacco users are presented in Table 3.

The variables were entered into the adjusted logistic regression model to find the most related factors for tobacco use (Table 4). Age (OR = 0.98; 95% CI: 0.98–0.99) and BMI (OR = 0.99; 95% CI: 0.98–0.99) were factors most related to tobacco use. Among socioeconomic related factors, lack of academic education (OR = 2.22; 95% CI: 1.73–2.86), (OR = 2.31; 95% CI: 1.91–2.80), (OR = 1.61; 95% CI: 1.33–1.95), having a manual job (OR = 1.55; 95% CI: 1.23–1.94), and low income level (OR = 1.63; 95% CI: 1.07–2.48) were the most significant factors associated with tobacco use.

**TABLE 4:** Related factors of tobacco use in Iranian adults based on OR and 95%CI using multiple logistic regression analysis

Variables	*B*	Adjusted OR (95% CI)	*P* value
Age	−0.019	0.98 (0.98–0.99)	<0.001
BMI	−0.014	0.99 (0.98–1)	0.023
Gender			
Women	Baseline	‐	‐
Men	0.068	1.07 (0.87–1.32)	0.524
Occupation			
Non‐manual	Baseline	‐	‐
Manual	0.439	1.55 (1.23–1.94)	<0.001
Jobless	0.355	1.42 (0.96–2.11)	0.076
Unspecified	0.105	1.11 (0.88–1.40)	0.374
Education			
Academic	Baseline	‐	‐
Illiterate	0.799	2.22 (1.73–2.86)	<0.001
Primary	0.836	2.31 (1.91–2.80)	<0.001
Secondary	0.477	1.61 (1.33–1.95)	<0.001
Income			
More than 7000$	Baseline	‐	‐
Less than 3500$	0.488	1.63 (1.07–2.48)	0.023
3500$ to 4750$	0.233	1.26 (0.82–1.94)	0.291
5250$ to 7000$	0.39	1.48 (0.9–2.43)	0.125

## 4 DISCUSSION

The current study reports the prevalence of tobacco use and its related socio‐economic factors in the city of Shiraz, in the southwest of Iran, based on the 2015 SARDS. A negative association was observed between tobacco use and the socioeconomic status of the individuals.

### 4.1 Active cigarette smoker

The prevalence of cigarette smoking was significantly higher among men (27.9%) compared to women (1.0%) in all age groups. The highest prevalence of cigarette smoking was observed in men and women in the 41–50 years age group (15.2%), which was in line with the previous reports. The prevalence of cigarette smoking was reported to be 21.4% vs. 1.1% among men and women, respectively, in a study conducted by Meysamie et al.^18^ Based on the National STEPS Surveys 2006–2009, 20.2% of men and 0.8% of women were exclusive daily cigarette smokers.^6^ In both studies, the highest prevalence of cigarette smoking was observed among individuals in the 45–54 years age group. This pattern could be considered part of an increasing trend of NCDs in the future elderly population of Iran.

### 4.2 Active hookah smokers

Surprisingly, hookah smoking was twice more prevalent among women than men (10.8% vs. 5.3%), and the highest prevalence belonged to the youngest age group (20–30 years) in both genders. The discrepancies in the definition of hookah use in different studies made the comparisons difficult. However, based on the results of some studies, the prevalence of hookah smoking was higher among young adults and women in southern regions of Iran.^6^, ^19^, ^20^ The rates of hookah smoking in the present study were closer to those reported from neighboring Arab countries where the prevalence of hookah smoking ranged from 9% to 15%.^21^, ^22^, ^23^

Although both cigarette and hookah smoking are among major behavioral health‐related risk factors, however, the obtained results were indicative of different usage patterns. Some vulnerable groups including women and young adults in the south of Iran are more prone to hookah smoking. This can be related to many false beliefs concerning hookah smoking, while recent findings have reported higher adverse effects and greater health‐related risks associated with hookah smoking compared to cigarette smoking.^24^, ^25^ In 2005, WHO introduced the term “cigarette‐hookah equivalence.” It was revealed that the volume of smoke produced by one session of hookah smoking is equal to the smoke produce by100 cigarettes.^26^, ^27^

### 4.3 Dual use

The prevalence of dual use (i.e., smoking both cigarettes and hookah) was low; however, it was more common among men than women in younger age groups. This finding was in line with the results of another study that reported the prevalence of dual use to be 0.3% among adult population of Iran in 2009.^6^

### 4.4 Second‐hand smokers

The prevalence of second‐hand smoking was highest among the tobacco use category. The prevalence of second‐hand smoking was approximately five times higher among women than men (*P* < 0.001). The prevalence of second‐hand smoking in our study was highest among younger age groups, which was consistent with the results obtained by Varmaghani et al.^28^ The higher prevalence of second‐hand smoking among women at home had been shown in a previous study.^29^

### 4.5 Tobacco use and socioeconomic related factors

According to univariate analysis, there were significant strong associations between tobacco use and almost all socio‐economic variables (*P* < 0.001). In adjusted multivariate logistic regression analysis, lack of academic education and having a manual job and a low‐income level were significant socioeconomic factors related to tobacco use. Based on the study performed by Hamrah et al.,^30^ the overall prevalence of tobacco use was 11.3% among the adult population of Shahroud City, Iran, where unemployment was one of the significant predictors of tobacco use. Although socioeconomic associations of tobacco use have not been investigated well until now, the role of social determinates of health, especially socio‐economic status, is crucial in fostering most behavioral risk factors. Therefore, attention to socioeconomic factors along with the existing tobacco control efforts can be useful in better control of morbidity and mortality of NCDs.

### 4.6 Strengths and limitations

The strengths of the present study include a large sample size from the population of the most crowded city in southern Iran. In addition, the use of a standard approach to determine the prevalence of tobacco use with respect to socioeconomic factors highlights the importance of social determinants of health in Iran. It is worth mentioning that the questionnaires were completed by a team of trained interviewers to increase the response rate. However, regarding the limitations of the present study, one can refer to the reliance on self‐reported income level as an indicator for the socioeconomic status of participants, which could have led to underreporting due to the social undesirability of this variable in the cultural setting of this area.^31^

The prevalence of tobacco use is high among Iranian adults in the southwest of Iran. Tobacco control efforts should be focused on more vulnerable groups of cigarette and hookah users. This study provides baseline information to highlight the effect of major social determinants of health on tobacco use. Further epidemiological studies would provide more evidence in this regard which can pave the way for the reduction of health‐related disparities and inequalities.

## CONFLICT OF INTEREST

The authors declare that they have no conflict of interest regarding the publication of this study.

## ETHICS STATEMENT

The study protocol was approved by the local Ethics Committee of Shiraz University of Medical Sciences, Shiraz, Iran (IR.SUMS.MED.REC.1398.122). Verbal informed consent was obtained from all the participants, and they were ensured about the confidentiality of their personal information.

## AUTHOR CONTRIBUTIONS

Mahdaviazad, Masoompour, and Foroutan were contributed in study design and acquisition, analysis and interpretation of data. Mahdaviazad and Foroutan were contributed in manuscript drafting. Masoompour critical revised the draft of manuscript. All authors read and approved the final manuscript.

## Data Availability

The data that support the findings of this study are available on request from the corresponding author. The data are not publicly available due to privacy or ethical restrictions.
